# Molecular diagnostic assays for the detection of common bacterial meningitis pathogens: A narrative review

**DOI:** 10.1016/j.ebiom.2021.103274

**Published:** 2021-03-12

**Authors:** Kanny Diallo, Vitalis F. Feteh, Lilian Ibe, Martin Antonio, Dominique A. Caugant, Mignon du Plessis, Ala-Eddine Deghmane, Ian M. Feavers, Katya Fernandez, LeAnne M. Fox, Charlene M.C. Rodrigues, Olivier Ronveaux, Muhamed-Kheir Taha, Xin Wang, Angela B. Brueggemann, Martin C.J. Maiden, Odile B. Harrison

**Affiliations:** a Department of Zoology, University of Oxford, South Parks Rd, Oxford OX1 3SY, United Kingdom; b Centre Suisse de Recherches Scientifiques en Côte d'Ivoire, Abidjan, Cote d'Ivoire; c WHO Collaborating Centre for New Vaccines Surveillance, Medical Research Council Unit The Gambia at London School of Hygiene & Tropical Medicine, Atlantic Boulevard, Fajara, PO Box 273, Banjul, Gambia; d WHO Collaborating Center for Reference and Research on Meningococci, Norwegian Institute of Public Health, Oslo N-0213, Norway; e A division of the National Health Laboratory Service (NHLS), National Institute for Communicable Diseases (NICD), Johannesburg, South Africa; f Institut Pasteur, 25-28 Rue du Dr Roux, Paris 75015, France; g WHO Infectious Hazard Management, Geneva, Switzerland; h National Center for Immunization and Respiratory Diseases, Centers for Disease Control and Prevention, Division of Bacterial Diseases, Meningitis and Vaccine Preventable Diseases Branch, United States; i Department of Paediatric Infectious Diseases, St George's University Hospitals NHS Foundation Trust, London, United Kingdom; j Nuffield Department of Population Health, Big Data Institute, University of Oxford, Oxford OX3 7LF, United Kingdom; k Department of Infection Biology, Faculty of Infectious and Tropical Diseases, London School of Hygiene & Tropical Medicine, London, United Kingdom

**Keywords:** Meningitis, Bacteria, Molecular diagnostics, PCR, RT-PCR, LAMP assay, Whole Genome Sequence data

## Abstract

Bacterial meningitis is a major global cause of morbidity and mortality. Rapid identification of the aetiological agent of meningitis is essential for clinical and public health management and disease prevention given the wide range of pathogens that cause the clinical syndrome and the availability of vaccines that protect against some, but not all, of these. Since microbiological culture is complex, slow, and often impacted by prior antimicrobial treatment of the patient, molecular diagnostic assays have been developed for bacterial detection. Distinguishing between meningitis caused by *Neisseria meningitidis* (meningococcus), *Streptococcus pneumoniae* (pneumococcus), *Haemophilus influenzae*, and *Streptococcus agalactiae* and identifying their polysaccharide capsules is especially important. Here, we review methods used in the identification of these bacteria, providing an up-to-date account of available assays, allowing clinicians and diagnostic laboratories to make informed decisions about which assays to use.

## 1 Introduction

Bacterial meningitis, which can be accompanied by sepsis, is an infection causing significant morbidity and mortality worldwide [1]. Many pathogens can invade the membranes lining the brain and spinal cord and cause syndromic meningitis; however, the condition can become rapidly fatal if untreated when caused by the encapsulated bacteria *Haemophilus influenzae, Neisseria meningitidis* (meningococcus)*, Streptococcus pneumoniae* (pneumococcus)*,* and *Streptococcus agalactiae* (group B streptococci, GBS)*.* In adults, older children, and adolescents (aged 10–19 years) bacterial meningitis caused by these organisms typically presents with symptoms including headache, fever, photophobia, vomiting, and neck stiffness [2]. In newborns (1 to 28 days), infants (up to 12 months) and young children (from 1 to 10 years), the symptoms and signs are non-specific, including lethargy, poor feeding, vomiting and irritability associated with fever [3]. Rapid, accurate and specific identification of the causative organism is necessary to ensure an effective public health response is elicited and appropriate clinical management, such as antimicrobial prophylaxis, can be established with or without vaccination of contacts.

The development and implementation of conjugate polysaccharide vaccines at the turn of the century transformed public health management of bacterial meningitis in developed countries, by providing vaccines that elicited individual protection and herd immunity against three of the most common causes of meningitis: the meningococcus; the pneumococcus; and *H. influenzae* (Table 1) [4]. Each polysaccharide vaccine, however, generates a highly specific immune response only to the polysaccharide antigens it contains. In the case of *H. influenzae*, one capsular type known as serotype b (Hib) is more commonly associated with causing disease, and monovalent vaccines against Hib have been highly successful. However, other serotypes (such as serotype a) are increasing in North America and broader vaccine development may be required [5]. For the meningococcus, six of the twelve capsular groups (known as serogroups *A, B, C, W, X*, and *Y*) cause the majority of meningococcal invasive disease. Conjugate vaccines are available for *A, C, W*, and *Y*: a serogroup X conjugate vaccine is in clinical trials, and alternative protein-based vaccines are available which protect against a number of meningococci including some that express serogroup *B* capsules [6].

**Table 1:** Current status of vaccines against H*. influenzae, N. meningitidis, S. pneumoniae* and *S. agalactiae*.

Bacterial species	Licensed vaccines and targets	Vaccines in clinical development	References
*Haemophilus influenzae*	ActHIB, Sanofi Pasteur Hib (PRP-T) Hiberix, GSK Vaccines Hib (PRP-T) PedvaxHIB, Merck Hib (PRP-OMP) ***Combination vaccines:*** Pentacel/Pentaxim, Sanofi Pasteur (DTaP, IPV, Hib [PRP-T]) Hexaxim/Hexyon, Sanofi Pasteur (DTaP, IPV, Hib [PRP-T], HBV) Vaxelis, Sanofi (DTaP, IPV, Hib [PRP-OMP], HBV) Infanrix Penta, GSK Vaccines (DTaP, IPV, Hib [PRP-T]) Infanrix Hexa, GSK Vaccines (DTaP, IPV, Hib [PRP-T], HBV) Menitorix, GSK Vaccines (Hib [PRP-T], MenC [PsC-T]) Menhibrix, GSK Vaccines (Hib [PRP-T], MenCY [PsC-T, PsY-T])		[84]
*Neisseria meningitidis*	***Polysaccharide vaccines:*** Menomune, Sanofi Pasteur (PsA, PsC, PsW, PsY) Mencevax/ACWYVax, Pfizer (PsA, PsC, PsW, PsY) NmVac4-A/C/Y/W-135, JN-International Medical Corporation (ACWY)		[1, 85]
	***Polysaccharide-protein conjugate vaccines:*** Menactra, Sanofi Pasteur (PsA-D, PsC-D, PsW-D, PsY-D) Menveo, GSK Vaccines (PsA-CRM197, PsC-CRM197, PsW-CRM197, PsT-CRM197) Mejugate, GSK Vaccines (PsC-CRM197) Nimenrix, Pfizer (PsA-T, PsC-T, PsW-T, PsY-T) NeisVac-C, Pfizer (PsC-T) MenAfriVac, Serum Institute of India (PsA-T) Menitorix, GSK Vaccines (Hib [PRP-T], MenC [PsC-T]) Menhibrix, GSK Vaccines (Hib [PRP-T], MenCY [PsC-T, PsY-T]) MenQuadfi, Sanofi Pasteur (MenACWY, [PRP-T])	***Polysaccharide-protein conjugate vaccines:*** NmCV-5, Serum Institute of India (PsA-T, PsC—CRM197, PsW-CRM197, PsX-T, PsY-CRM197)	
	**Outer membrane vesicle vaccines:** VA-MENGOC-BC, Finlay Institute Vaccine, Cuba (CU385/83 B:4:P1.19, 15) (no longer in production)		
	**Protein-based vaccines:** Bexsero (4CMenB), GSK Vaccines (NZ98/254 OMV, FHbp, NadA, NHPA) Trumenba, Pfizer (bivalent FHbp)		
*Streptococcus pneumoniae*	**Polysaccharide vaccine:** Pneumovax®23 - serotypes: 1, 2, 3, 4, 5, 6B, 7F, 8, 9 V, 9 N, 10A, 11A, 12F, 14, 15B, 17F, 18C, 19F, 19A, 20, 22F, 23F, 33F		[86]
	**Polysaccharide-protein conjugate vaccines:** Synflorix™, GSK Vaccines (10-valent) – serotypes: 1, 4, 5, 6B, 7F, 9 V, 14, 18C, 19F, 23F Prevenar/Prevnar®, Pfizer (13-valent) – serotypes: 1, 3, 4, 5, 6A, 6B, 7F, 9 V, 14, 18C, 19A, 19F, 23F Pneumosil, Serum Institute of India (10-valent) – serotypes: 1, 5, 6A, 6B, 7F, 9 V, 14, 19A, 19F, 23F	**Polysaccharide-protein conjugate vaccines in Phase 3 clinical trials:** V114, MSD (15-valent) – serotypes 1, 3, 4, 5, 6A, 6B, 7F, 9 V, 14, 18C, 19A, 19F, 22F, 23F, and 33F 20vPnC, Pfizer (20-valent) – serotypes: 1, 3, 4, 5, 6A, 6B, 7F, 8, 9 V, 10A, 11A, 12F, 14, 15B, 18C, 19A, 19F, 22F, 23F, 33F	
*Streptococcus agalactiae*	Licenced vaccines not yet available	Pfizer: Up to 6-valent vaccine recruiting for trials Minervax: N-terminal domains of the Rib and AlphaC surface protein vaccines	[7]

For the pneumococcus the challenge is greater, since there are a large number of capsular serotypes associated with invasive pneumococcal disease and current vaccines only cover up to 13 of these (Table 2). Furthermore, although there are vaccines under development, no licenced vaccine against GBS was available at the time of writing [7]. Notwithstanding these limitations, the widespread implementation of available conjugate vaccines has resulted in a range of public health benefits including: (i) The global decrease of invasive disease due to Hib [8]; (ii) The decrease in serogroup C meningococcal disease in countries that implemented the serogroup C conjugate vaccine; (iii) The near disappearance of serogroup A meningococcal infections in the African meningitis belt; and (iv) Major reductions in invasive pneumococcal disease globally [4,[9], [10], [11]].

**Table 2:** Known capsular polysaccharides among the four major bacterial meningeal pathogens.

Bacterial species	Known capsular types
*Haemophilus influenzae*	a b, c, d, e, f
*Neisseria meningitidis*	A, B, C, E, H, I, K, L, W, X, Y and Z
*Streptococcus pneumoniae*	1, 2, 3, 4, 5, 6A, 6B, 6Bii (6E), 6C, 6D, 6F, 6 G, 6H, 7F, 7A, 7B, 7C, 8, 9A, 9 L, 9 N, 9 V, 10F, 10A, 10B, 10C, 11F, 11A, 11B, 11C, 11D, 11E, 12F, 12A, 12B, 13, 14, 15F, 15A, 15B/C, 16F, 16A, 17F, 17A, 18F, 18A, 18B, 18C, 19F, 19A, 19B, 19C, 20A, 20B, 21, 22F, 22A, 23F, 23A, 23B, 24F, 24A, 24B, 25F, 25A, 27, 28F, 28A, 29, 31, 32F, 32A, 33F, 33A, 33B, 33C, 33D, 33E, 34, 35F, 35A, 35B, 35C, 36, 37, 38, 39, 40, 41F, 41A, 42, 43, 44, 45, 46, 47F, 47A, 48
*Streptococcus agalactiae*	Ia, Ib, II, III, IV, V, VI, VII, VIII, IX

**Fig. 1:**
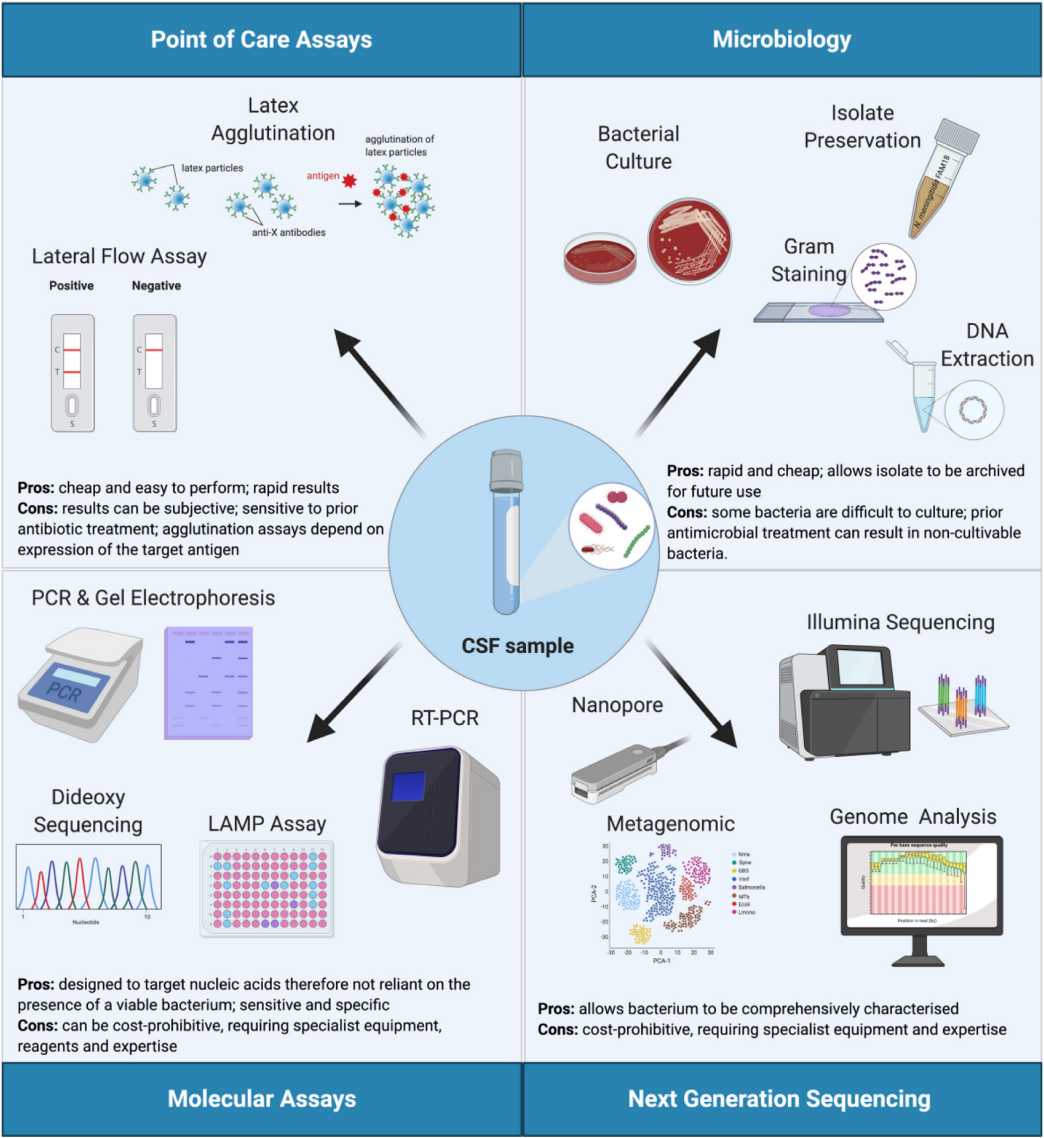
Approaches used in the diagnosis of meningitis from cerebrospinal fluid. Figure created with BioRender.com.

Given the diversity of organisms that can cause meningitis, accurate species and capsule identification is essential for diagnosis, treatment, surveillance, and public health intervention. This ensures that relevant measures are implemented for case and outbreak management and also informs future vaccine development. A global initiative aimed at eliminating meningitis worldwide by 2030 has recently been endorsed by the WHO (https://www.who.int/activities/defeating-meningitis-by-2030). One of five pillars of the roadmap for this global vision is “Diagnosis and treatment” and it highlights the need for comprehensive, cost-effective diagnostic approaches to enhance surveillance [12]. A number of diagnostic assays have been developed (Fig. 1). Here, we provide an account of recently published molecular diagnostic assays and report on the developments made in this field. Clinicians and diagnostic laboratories can use this information to make decisions on assay suitability and identify areas that require further research.

### 1.1 Point-of-care assays

Characterisation of organisms by culture from a normally sterile site, such as cerebrospinal fluid (CSF), has long been the mainstay of the laboratory confirmation of suspected bacterial meningitis [2]. However, several factors impede the use of CSF culture for diagnosis, including: the fact that it can take 24–48 h or longer to obtain culture results [13]; suboptimal CSF specimen volume; fastidious, difficult to cultivate organisms; suboptimal storage and transportation; and prior antimicrobial administration resulting in non-viable bacteria. Consequently, a variety of approaches have been developed for the diagnosis of bacterial meningitis, including: latex agglutination, lateral flow assays, and molecular diagnostic assays (Fig. 1).

Although sero-agglutination assays are among the simplest tests to execute, their sensitivity can be reduced if the patient received antimicrobial treatment prior to specimen collection [14]. Agglutination assays also depend on expression of the target antigen, most often the polysaccharide capsule which bacteria can modulate both *in vitro* and *in vivo* [15,16]. Cross-reactivity due to poly-agglutination can be problematic for some sero-agglutination assays and can prevent definitive characterisation of the isolate [17]. Lateral flow assays (LFAs; one example is the commercial pregnancy test), consist of prefabricated strips containing dry reagents that are activated by application of a fluid sample [18], [19], [20]. Several types of LFAs exist and the greatest benefits of these tests are that they are rapid, single use, and point-of-care. The different types of LFAs include those in which antibodies are used as recognition elements (‘lateral flow immunoassays’). An example of this is the MeningoSpeed rapid diagnostic test, an immunochromatographic test that can detect meningococcal serogroups from CSF samples [21]. Other LFAs consist of antigen-antibody interactions with specific tagged doubled-stranded amplicon detection after PCR (‘nucleic acid lateral flow immunoassay’) or assays where specific nucleic acid amplicons are hybridised with immobilised complementary probes (‘nucleic acid lateral flow assay’) [20]. Although the sensitivity of sero-agglutination and LFAs is susceptible to bacterial viability, the ease and rapidity with which these assays can be used make these assays desirable in more challenging low- and middle-income countries (LMIC).

### 1.2 Endpoint PCR and real-time PCR assays for the detection of meningitis pathogens

Molecular tools including the polymerase chain reaction (PCR), real-time PCR (rtPCR), qualitative or quantitative PCR (qPCR), and loop-mediated isothermal amplification assays (LAMP) have the potential to overcome many of the limitations of culture-based approaches, as they target bacterial DNA and are not constrained by the presence of cultivable organisms. These molecular tools are now the methods of choice for many laboratories and have improved public health measures because they involve standard, generic laboratory techniques that allow multiple pathogens to be rapidly detected. Indeed, the World Health Organisation (WHO) recommends the use of rtPCR in the testing of pneumococcus, meningococcus, and Hib from suspected cases of meningitis [13]. Major challenges remain, however, in the deployment of these assays in LMICs, due to the variability of laboratory capabilities, shortage of trained laboratory personnel and challenges in procurement of reagents and equipment. Progress has been made through initiatives such as MenAfriNet (www.menafrinet.org) and laboratories in several countries in the African meningitis belt and beyond have acquired the capability to perform these assays [22].

The first molecular methods to be developed were PCR assays that detect a single pathogen, but these have been largely superseded by multiplex PCR assays (simultaneous testing of multiple targets) such as rtPCR assays, which reduces time and consumable costs [23]. Note that the tests described below are not commercially available except where indicated.

#### 1.2.1 Haemophilus influenzae

Several singleplex PCR assays detecting Hib have been described, including those that detect: the capsule gene *bexA* [24]; the outer membrane proteins OmpP2 (*ompP2*) and OmpP6 (*ompP6*) [25,26]; the phase variable gene *licA* encoding phosphorylcholine kinase [25]; the protein D gene (*hpd*) [26,27]; and fuculokinase (*fucK*) [25] (Table 3). Although assays targeting *fucK* and *hpd* have been shown to reliably detect Hib [25,28], some non-typeable *H. influenzae* (NTHi) strains have been reported to lack either *fucK* [29] or *hpd* genes [30] while some *Haemophilus parainfluenzae* strains were reported to possess *hpd* [28] (Table 3). The *ompP2* and *ompP6* sequences also display some diversity, making them difficult targets to detect reliably, and there is some evidence for cross-reactivity with *Haemophilus haemolyticus* [31]. The increasing availability of whole genome sequence data (WGS) will provide further opportunities to determine the prevalence, diversity and distribution of these genes within the genus *Haemophilus.*

**Table 3:** Genetic targets used in the detection of the four main causes of bacterial meningitis.

Bacterial species	Genetic target and type of assay	Function	Disadvantage (if known)	Sensitivity (%)	Specificity (%)	References
*Haemophilus influenzae* (Hib)	**rtPCR**					
	*bexA*	ATP-binding protein (capsule region I)	Does not detect NTHi strains	100	90–100	[21]
	*omP2*	Outer membrane protein P2	Less sensitive than *fucK* or *licA*, and requires very high genome copies for detection	97.1	100	[25]
	*omP6*	Outer membrane protein P6	Nucleotide sequence diversity	NA	NA	[31]
	*licA*	Protein LicA	Fails to detect some serotype e isolates	97.1	99.1	[25]
	*hpd*	Protein D	May be absent in some NTHi strains	95/95.7	91/ 92.3	[30]
	*fucK*	Fuculokinase	Deletion of the fucose operon in some strains and reactivity with *H. aegyptius*	97.1	100	[29]
	**LAMP:**					
	Hib capsule	247 bp region of the capsule		100	100	[62]
	*pstA*	Phosphate transport system permease protein		80	100	[68]
*Neisseria meningitidis* (meningococcus)	**rtPCR**:					
	*ctrA*	Capsule transport (region I capsule locus)	Only encapsulated meningococci have *ctrA*	71.6	NA	[33]
	*sodC*	Superoxide dismutase	Less sensitive in sterile body fluids, for use in conjunction with *ctrA* to improve sensitivity Some species possess homologous *sodC* genes	99.6/ 94.7	100/ 77.9	[32]
	*crgA*	Transcriptional regulator of LysR family	Can be found in *N. gonorrhoeae*	93	96	[87]
	*porA*	Outer membrane porin, PorA	Can be found in *N. gonorrhoeae* and may be absent in some meningococci	96.1	91.6	[88,89]
	**LAMP:**					
	*ctrA*	Capsule transport		89/100	100/98.9	[64,65]
	*NMO_1242*	Putative cytolysin secretion ABC transporter protein		100	100	[68]
*Streptococcus pneumoniae* (pneumococcus)	**rtPCR:**					
	*lytA*	Autolysin	Possible homologues in other closely-related species leading to false positives	100	99.5/100	[36,38]
	*piaB*	Pia ABC transporter	*piaB* is absent in some pneumococci including some serotype 6B strains and absent in some non-typeable strains	95.3	99.5	[36]
	GntR-family SP2020	Putative transcriptional regulator gene	May be present in non-pneumococcal strains; combining with *lytA* results increases sensitivity	100	99.8	[36]
	*ply*	Pneumolysin	Can lead to false-positive reactions in the presence of viridans group streptococci	100	81	[38]
	**LAMP:**					
	*lytA*	Autolysin		100	100	[66]
	*SPNA45_01710*	Heparinase II/III-like protein		95.7	100	[68]
*Streptococcus agalactiae* (GBS)	**rtPCR:**					
	*cfb*	*S. agalactiae* CAMP factor	some CAMP-negative GBS may not carry the *cfb* gene	NA	NA	[41]
	*dltS*	Histidine kinase specific to GBS		96.1	95.9	[59]
	**LAMP:**					
	*cfb*	*S. agalactiae* CAMP factor		NA	NA	[41]

#### 1.2.2 Neisseria meningitidis

Recommended genetic targets for the identification of meningococci include the superoxide dismutase gene *sodC* and the capsule gene *ctrA* (Table 3) [32,33]. Although *sodC* is ubiquitous in meningococci, it has been reported to lead to false positive results due to the presence of *sodC* homologues in other bacterial species including *H. influenzae* [33], while the *ctrA* gene preferentially detects encapsulated meningococci (Table 3). As a result, other tests have been developed, using the outer membrane protein gene *porA* and the capsule null locus (*cnl*), the combination of which identifies unencapsulated meningococci, which cause invasive disease rarely, but which are commonly found in asymptomatic carriage [33,34]. In such situations, the host may have a predisposing immunosuppressive condition [35]; however, this also suggests that rtPCR assays that only test for encapsulated meningococci may be overlooking *cnl* strains, and that a combined *cnl* and *ctrA* assay may be needed to increase sensitivity.

#### 1.2.3 Streptococcus pneumoniae

The genetic targets used to detect *S. pneumoniae* include the autolysin gene *lytA* and the permease gene of the Pia ABC transporter, *piaB,* although the latter was reported to be absent in some nontypeable or serotype 6B pneumococci [36]. Consequently, *lytA* was the recommended genetic target for pneumococci at the time of writing [36]. It has been reported that *lytA* homologues can be present in closely-related species of *Streptococcus* [37], therefore an assay targeting a putative transcriptional regulator of the GntR-family (gene SP2020), belonging to the core genome of the pneumococcus, was developed [36] (Table 3). Finally, the pneumolysin gene, *ply*, as well as the pneumococcal surface adhesion gene, *psaA*, have been described as targets for the detection of pneumococci in rtPCR assays; however, strains of *Streptococcus pseudopneumoniae* and viridans group streptococci can also be positive for these genes, precluding their use as reliable genetic targets [38].

#### 1.2.4 Streptococcus agalactiae

Singleplex assays detecting GBS have also been developed, although many of these are optimised for identifying the bacterium from gut or vaginal colonisation and/or shortly after labour rather than for the diagnosis of acute infection [39,40]. Four commercial assays are available: (i) The Becton Dickinson MAX GBS assay; (ii) The ARIES GBS assay from Luminex Corporation; (iii) The Illumigene Group B Streptococcus assay from Meridian Bioscience; and (iv) The Xpert GBS LB assay produced by Cepheid Inc. Three of these use rtPCR assays, while Illumigene uses a loop-mediated isothermal amplification (LAMP) assay with the *cfb* gene as the primary target. The gene *cfb* encodes the extracellular pore-forming toxin also known as the CAMP factor [40]. The Christie-Atkinson-Munch-Peterson (CAMP) test has been the conventional culture-based test for identifying GBS and differentiates haemolytic versus non-haemolytic GBS. Notably, some GBS isolates are CAMP negative and lack *cfb*, indicating that further studies assessing the distribution of *cfb* are required [41,42]. Another assay employed a recombinase polymerase amplification (RPA) method to detect the *cfb* gene in vaginal swabs and this assay was sensitive and specific for GBS [43]. All these assays required samples to be enriched in Lim Broth for 18–24 h prior to testing and results demonstrated greater sensitivity compared with culture alone.

### 1.3 Multiplex assays

An advantage of using PCR assays in infectious disease diagnostics is that multiple pathogens may be targeted in a single assay, which conserves clinical specimens, saves time, and reduces costs. Multiplex rtPCR assays that detect Hib, meningococci and pneumococci in one reaction have been developed and can be used to test CSF directly and avoid the need for DNA extraction [44,45]. One prototype assay has been developed to detect six microorganisms associated with meningitis including (molecular target): *S. pneumoniae* (*lytA*); *N. meningitidis (ctrA); H. influenzae (ompP2); S. agalactiae (cfb); Listeria monocytogenes (iap)*; and *Cryptococcus neoformans* (18S rDNA) [46]. To date, the assay has been tested using a limited number of specimens and therefore requires further validation; however, of the 45 suspected cases of bacterial meningitis, the causative agent was identified in 32 CSF specimens using a combination of phenotypic and genotypic tests and of these 16 were identified solely on the basis of molecular assays [46].

More recently, a commercial TaqMan Array card has been developed that can detect multiple meningitis-associated pathogens [47]. This assay was tested in CSF samples originating from West Africa and results indicated that diverse infectious aetiological agents were present. Another commercially available test is the BioFire® FilmArray® Meningitis/Encephalitis (ME) Panel which can detect 14 pathogens (seven viruses, six bacteria and one fungus) simultaneously from CSF samples, including the four bacteria that are the focus of this review [48]. Performance of the ME Panel was evaluated using clinical CSF samples that had previously tested positive using routine methods: the ME Panel resulted in an overall positive agreement of 97.5% for bacterial pathogens [48]. A study in Ethiopia involving 218 patients with suspected meningitis identified *S. pneumoniae, N. meningitidis* and *H. influenzae* using the ME Panel, but only in 5 (2.2%) cases [49]*.* Although the ME Panel has been shown to increase pathogen detection rate, it has been noted that interpretation and correlation of results requires experienced users and it does not allow the capsular polysaccharide to be determined [50]. Furthermore, the ME Panel may be cost-prohibitive for LMICs since the estimated average cost is currently around $239 per test (around £180/€200) [51].

It should also be noted that there are several other aetiological agents of meningitis, some of which are highly prevalent in LMIC settings, including *Cryptococcus neoformans; Mycobacterium tuberculosis; Salmonella enterica var* Typhi*;* Herpes simplex virus; Varicella zoster virus and enteroviruses. *C. neoformans* and *M. tuberculosis* in particular are common among HIV-infected individuals. Inexpensive, easy to use multiplex assays with the capacity to detect all of these pathogens need to be developed.

### 1.4 PCR and rtPCR assays for the detection of capsular types

Once the bacterial species has been confirmed, further PCR assays are available to detect capsule types, which is important in assessing capsule-specific disease burden and guiding vaccination decisions. Given that the majority of invasive disease cases caused by *H. influenzae* are due to serotype b, few assays detecting the remaining five capsules have been developed, although a PCR-endpoint based assay can be used to detect *H. influenzae* serotypes [52,53]. Multiple capsular polysaccharides are associated with invasive disease and, differentiation of these capsules is essential (Table 2). Assays have been developed that detect meningococcal or pneumococcal capsules associated with invasive disease [45,54]. Whilst rtPCR assays to detect meningococcal serogroups *A, B, C, W, Y*, and *X* have been developed, the complexity of the pneumococcal capsular locus and large number of serotypes make the design of multiple PCR assays capable of detecting a large number of potential serotypes a major challenge [55,56]. Most assays focus on detecting a subset of the prevalent serotypes circulating in a region and/or those most frequently associated with invasive disease. An alternative approach determines serotypes based on the capsule polymerase gene *wzh* [57].

There are ten GBS serotypes and six serotypes (Ia, Ib, II, III, IV and V), in particular, are associated with invasive disease. Few rtPCR assays have been designed to detect the presence of *S. agalactiae* with one based on the *dltS* gene, a sensor protein, and capsule-specific genes encoding serotypes Ia, Ib, and III [58,59]. The assay is species-specific for GBS with no cross-reaction with other closely related *Streptococcus* spp and it reliably detects serotypes Ia, Ib, and III.

### 1.5 Loop-mediated isothermal amplification assays (LAMP)

The loop-mediated isothermal amplification (LAMP) assay was developed in 2000 [60]. This assay amplifies a specific DNA target and has the advantage of working in isothermal conditions, removing the requirement for thermocyclers. LAMP uses a DNA polymerase that has strand displacement activity, allowing it to separate the double-stranded DNA without the need for a temperature change and thus the reaction can be conducted in a simple water bath. In addition to requiring very basic equipment, LAMP assays have been found to be highly specific [61] and their efficiency is less affected by background DNA [60]. As a result, several LAMP assays have been designed for the diagnosis of bacterial pathogens, including the four pathogens included in this review.

A LAMP assay to detect Hib directly from CSF was described in 2011 [62], which targeted a 247 bp nucleotide sequence of the capsule locus and was performed at 65 °C for 35 min. The method was evaluated using a collection of *H. influenzae* isolates, other *Haemophilus* species, and other genera, as well as stored CSF samples originating from suspected meningitis cases. The Hib LAMP assay discriminated Hib from other encapsulated *H. influenzae* strains and was more sensitive than the *bexA* PCR assay and a nested PCR for the detection of Hib [62]. Another study used a similar design to develop five LAMP assays for the characterisation of non-serotype b *H. influenzae* isolates [63]. Validation was performed using a collection of *H. influenzae* isolates, other *Haemophilus* species, other genera, and spiked CSF samples. This non-serotype b LAMP assay was as specific and sensitive as the comparable non-serotype b PCR assay, and results were confirmed by dideoxy nucleotide sequencing of the products [63].

A LAMP assay for the detection of *N. meningitidis* uses primers that target the *ctrA* gene. This assay performed as well as the standard rtPCR, but at a fraction of the cost and time [64]. The method was assessed as a point-of-care diagnostic tool at the emergency department of the Royal Belfast Hospital for Sick Children, UK [65]: A total of 161 patients had nasopharyngeal and blood samples tested using the LAMP assay in addition to the routine culture and PCR but the assay was not validated for CSF specimens. Similarly, a LAMP assay targeting the *lytA* gene of *S. pneumoniae* was developed and validated using a set of reference strains that included different *Streptococcus* species and other genera, clinical alpha-haemolytic streptococcal isolates, and CSF samples from suspected meningitis cases [66]. The *lytA* LAMP assay was found to be more sensitive than the comparable *lytA* PCR.

Multiplex LAMP assays have been more difficult to design because of the non-exonuclease activity of the polymerase enzyme, which prevents the use of labelled probes; however, the ability to detect multiple pathogens in a single reaction has been explored in the context of meningitis. A prototype LAMP assay was designed to detect *S. pneumoniae, Staphylococcus aureus, Streptococcus suis,* and GBS based on the sequence diversity of the 16 rRNA genes of each bacterial species [67], which exhibited better sensitivity and a lower limit of detection than conventional PCR. It was specific, although only three other bacterial species were tested for cross-reactivity (*N. meningitidis, H. influenzae*, and *Escherichia coli*). The assay was validated with DNA extracted from cultured isolates but was not tested on CSF.

A modified LAMP assay, the Tth Endonuclease Cleavage (TEC) LAMP, has been developed, allowing simultaneous detection of *N. meningitidis, S. pneumoniae,* and *H. influenzae* [68]. This assay includes a thermostable enzyme capable of cleaving abasic sites in double-stranded DNA, along with a TEC primer/probe, which acts as a LAMP forward inner primer, but which also contains an abasic site, a fluorescent dye, and a quencher [68]. The addition of the fluorescent dye allows multiplexing and real-time monitoring of the amplification. The assay targets the genes encoding: (i) A heparinase II/III-like protein in *S. pneumoniae* (*SPNA45_01710*); (ii) A putative cytolysin secretion ABC transporter protein in *N. meningitidis* (*NMO_1242; hylB*); and (iii) A phosphate transport system permease protein in Hib (*pstA*). A random gene fragment is incorporated as an internal control [68]. This prototype assay was validated using a panel of DNA extracted from reference and clinical isolates and tested in PCR-confirmed cases of *S. pneumoniae, N. meningitidis*, and *H. influenzae* infections. The specificity was 100% for each target and pathogen-specific sensitivity was 95.7% for *S. pneumoniae*, 100% for *N. meningitidis* and 80% for *H. influenzae*. Although the reactions were conducted in a rtPCR thermocycler, this assay could also be performed with simpler point-of-care technologies that allow sample heating and fluorescent detection [69]. An improvement of the (TEC)-LAMP method is loop-primer endonuclease cleavage (LEC)-LAMP, which allows single nucleotide polymorphisms to be detected in either singleplex or multiplex assays [70]. This assay successfully resulted in the simultaneous detection of *N. meningitidis, S. pneumoniae*, and *H. influenzae* and may be useful in the detection of allele-specific differences between and within bacterial species in a single assay. Finally, a commercial LAMP assay called the eazyplex® CSF direct panel is available and has the capacity to detect several pathogens associated with meningitis, including *E. coli, H. influenzae, L. monocytogenes, N. meningitidis, S. agalactiae,* and *S. pneumoniae*. The average cost of this assay was estimated to be around $62 (around £45/€50) per sample and was relatively easy to use with a sensitivity of 90.9% and specificity of 100% [71].

Ultimately, LAMP technology could be developed into affordable point-of-care devices such as paper-based LAMP assays that allow the simultaneous detection of *S. agalactiae, S. pneumoniae* and *S. aureus* using dry reagents that are easy to store and transport in LMICs [72]. Improvements in LAMP methodologies that could allow for real-time monitoring system without post-amplification manipulations make these assays potential candidates for the molecular diagnosis of infectious diseases such as meningitis. The pooled high sensitivity and specificity values determined from a review of LAMP assays detecting the meningococcus are encouraging [73] and the design of a multiplex LAMP assay including all four pathogens included in this review is now a possibility. Such an assay would be invaluable for use in hospital settings and the lack of requirements for expensive equipment makes it an appealing approach for laboratories with limited resources.

### 1.6 Sensitivities and specificities of published assays

The sensitivity and specificity values of various singleplex rtPCR assays were high, ranging from 91 to 100% (Table 3). Multiplex assays targeting *S. pneumoniae, N. meningitidis and H. influenzae* were reported to have sensitivities ranging from 73 to 94%, and good specificities (98 to 100%), comparable to those of singleplex rtPCR assays. LAMP assays were found to generate both higher sensitivity and specificity values compared to rtPCR (Table 3). The accuracy of LAMP assays was consistently high (sensitivity of 80–100% and specificity of 99–100%) using various clinical samples including CSF, blood and nasopharyngeal swabs from children.

Among PCR-based tests, the sensitivity and specificity values were higher for CSF samples than for either blood samples or oro-nasopharyngeal swabs for the diagnosis of meningitis; however, using LAMP assays that target *ctrA* loci for the diagnosis of invasive meningococcal disease, the sensitivity and specificity values were similar regardless of the sample used [65]. Nonetheless, further studies are needed to clearly elucidate whether other non-CSF specimens are suitable for LAMP diagnosis of meningococcal disease.

## 2 Outstanding questions

In the longer term, whole genome sequencing (WGS) will become more routinely employed in the diagnosis of infectious diseases, including meningitis [74]. Ultimately, this will likely include the development of non-culture-based methods that directly sequence pathogen genomes from clinical specimens. Current requirements for these approaches include DNA extraction protocols that deplete human DNA and increase the bacterial sequence yield [75]. Such assays will have the benefit of allowing multiple genetic targets to be analysed directly from genomes, enabling the simultaneous identification of the pathogen, the predicted capsular type, antimicrobial resistance determinants, etc. Progress has been made in this respect with selective whole-genome amplification (SWGA), an isothermal multiple-displacement amplification-based method, validated in 12 CSF specimens from invasive meningococcal disease cases [76]. In addition, metagenomic next generation sequencing is also showing promise in improving the diagnosis of meningitis and encephalitis [77,78]. Oxford Nanopore sequencing technology was recently employed in Zambia for the rapid diagnosis of bacterial meningitis species through the sequencing of 16S rRNA [79].

To reach the goal of implementing WGS in LMICs, a range of technical, financial, and infrastructural challenges will have to be met and, until then, it is unlikely laboratories in LMICs will be able to routinely undertake WGS for the diagnosis of meningitis. In the immediate future, the most promising opportunities involve refining molecular diagnostic assays using comparative genomic analyses to: (i) Improve practices of sampling, storage and transport of specimens; (ii) Improve the performance of existing targets by increasing sensitivity and specificity; and (iii) Identify alternative diagnostic targets to enhance species-specificity [80]. In order to undertake such activities, an enhanced representation of genomes originating from LMICs needs to be established and initiatives such as the Global Meningitis Partnership seek to address this issue [81]. Furthermore, the analysis and comparison of genomes relies on the availability of databases where genomes can be stored, curated and analysed. For example, PubMLST databases (https://pubmlst.org) store and annotate thousands of genomes (with associated metadata) of all four bacterial species included in this review in dedicated genome libraries [82] and BMGAP allows users to deposit and access *N. meningitidis* and *H. influenzae* genomes using a secure online portal [83].

The timely diagnosis of the aetiological agent in meningeal infections is essential to facilitate treatment, patient management, and improved clinical outcomes. The most desirable diagnostic assay would be one in which multiple pathogens can be detected in a cost-effective, easy to use system that provides rapid and robust results. This review has found that extensive progress has been made in the development of diagnostic methods for meningitis; however, it is likely that multiple approaches will still be required, and cost and ease of use are major influencing factors in the choice and utility of diagnostic tests.

### 2.1 Search strategy and selection criteria

Three independent reviewers searched for relevant publications dating from 2010 to 2020 using the Medical Subject Headings (MeSH): ‘molecular diagnostics’ AND ‘bacterial meningitis’; or ‘molecular diagnostics’ AND *‘Haemophilus influenzae’* or ‘*Neisseria meningitidis’* or ‘*Streptococcus pneumoniae’* or *‘Streptococcus agalactiae’*. The terms ‘Hib’ and ‘group B streptococcus’ were also included. The electronic literature databases Medline/Ovid, PubMed, Web of Science, Embase and Global Health were used. Assays designed to identify bacterial polysaccharide capsules were also selected. Publications describing viral, *Mycobacterium tuberculosis*, respiratory or animal infections, were excluded as were articles that: (i) Did not include full texts; (ii) Were duplicated; (iii) Described MALDI-ToF assays; or (iv) were not in English.

Sensitivity and specificity values were derived by determining True Positive (TP), False Positive (FP), False Negative (FN) and True Negative (TN) values from appropriate published assays. The reference ‘standard’ was determined to be a culture-based result and a molecular assay was the ‘test’ result.

## Contributors

All of the authors have read and approved the final version of the manuscript.

KD, LI, OBH performed literature reviews. KD, OBH, MCJM, ABB study design. KD, OBH, MCJM, ABB, MA, DAC, MdP, AD, IMF, KF, LMF, CMCR, OR, MKT, XW: contributed to writing

KD, OBH, VFF contributed to figures, data analysis and interpretation.

## Declaration of Competing Interest

The authors confirm there are no conflicts of interest.
